# Occurrence of Patellofemoral Joint Osteoarthritis in Long-Term Postoperative Cases of Open-Wedge High Tibial Osteotomy: Differences in Symptoms Based on Patient-Standing Type Evaluation with and Without Patellofemoral Joint Osteoarthritis

**DOI:** 10.1007/s43465-024-01250-z

**Published:** 2024-08-29

**Authors:** Noriyuki Gomi, Hiroaki Muramoto, Yusuke Kataoka

**Affiliations:** Department of Joint Surgery Center, Sakaide Kaisei General Hospital, 3-5-28 Muromachi, Sakaide City, Kagawa Japan

**Keywords:** Long-term course, Open-wedge high tibial osteotomy, Patellofemoral joint, Osteoarthritis, Patient-based evaluation, Symptoms

## Abstract

**Purpose:**

To examine the frequency of patellofemoral joint (PFJ) osteoarthritis (OA) and its symptoms in the long-term course of open-wedge high tibial osteotomy (OWHTO).

**Methods:**

We analyzed 113 joints of 91 patients. OA and osteonecrosis (ON) developed in 91 and 22 joints, after an average postoperative period of 127.5 ± 19.5 months. For X-ray evaluation, the standing femorotibial angle (FTA), % mechanical axis (%MA), Caton–Deschamps index (CDI), patellar tilt angle (TA), lateral patellar shift (LPS), and PFJ space width (medial [MJS] and lateral [LJS]) were analyzed. PFJ-associated symptoms were evaluated using the hospital for special surgery patellar score (HSS-PS) and knee injury and osteoarthritis outcome score patellofemoral subscale (KOOS-PF). Statistical analysis was performed with paired and unpaired t tests, and a risk rate of less than 1% was significantly judged.

**Results:**

Preoperative FTA and CDI decreased from 180.8° to 170.0° and 0.88 to 0.70 at the final follow-up. Preoperative %MA lateralized from 20.8 to 66.0 at the final follow-up. TA and LPS values decreased significantly compared with before surgery until plate removal. The MJS and LJS significantly decreased, and OA with a joint space < 3 mm occurred in 14 cases. However, HSS-PS and KOOS-PF scores were not significantly different between the groups with and without OA.

**Conclusion:**

PFJ OA occurred in 12.4% cases in the long-term postoperative course of OWHTO; however, no symptomatic difference was found in the group with or without OA.

**Supplementary Information:**

The online version contains supplementary material available at 10.1007/s43465-024-01250-z.

## Introduction

Open-wedge high tibial osteotomy (OWHTO) is performed from the proximal medial side of the tibial tuberosity till the vicinity of the proximal tibiofibular joint for correcting the knee in the valgus position by opening the osteotomy line. This procedure does not require a fibula osteotomy and provides excellent initial fixation through preservation of the lateral bone cortex and provision of strong internal fixation. OWHTO has become popular in recent years because it has the advantages of being a simpler procedure and having a shorter post-treatment period than conventional closing-wedge HTO [1, 2]. However, the load on the patellofemoral joint (PFJ) and osteoarthritis (OA) is a problem caused by the distal movements of the tibial tubercle [3–6]. To prevent this, distal tuberosity osteotomy (DTO), which leaves the tibial tubercle proximally, has also been reported [7, 8]. However, few studies have reported the changes and symptoms associated with the PFJ after OWHTO [9–11], and there are no reports of long-term progress. Thus, in this study we investigated the morphologic changes of the PFJ, OA onset, and associated symptoms during the long-term postoperative course of OWHTO.

## Materials and Methods

Patients who underwent OWHTO between June 2007 and April 2014 in our hospital were included. Patients whose last direct examination period was < 8 years were excluded. There were no age restrictions. OWHTO was indicated for patients with symptomatic medial OA or osteonecrosis (ON) with high activity, and the corrected angle was determined so that the postoperative % mechanical axis (%MA) was 65 in standing anteroposterior whole-leg radiographs.

In the study, we analyzed 113 joints in 91 patients, 28 joints in 21 male patients, and 85 joints in 70 female patients. The average age at the time of surgery was 64.4 ± 6.2 (49–78) years, 91 joints had OA, 22 joints had ON, and the average period after the operation was 127.5 ± 19.5 (97–171) months.

**Fig. 1:**
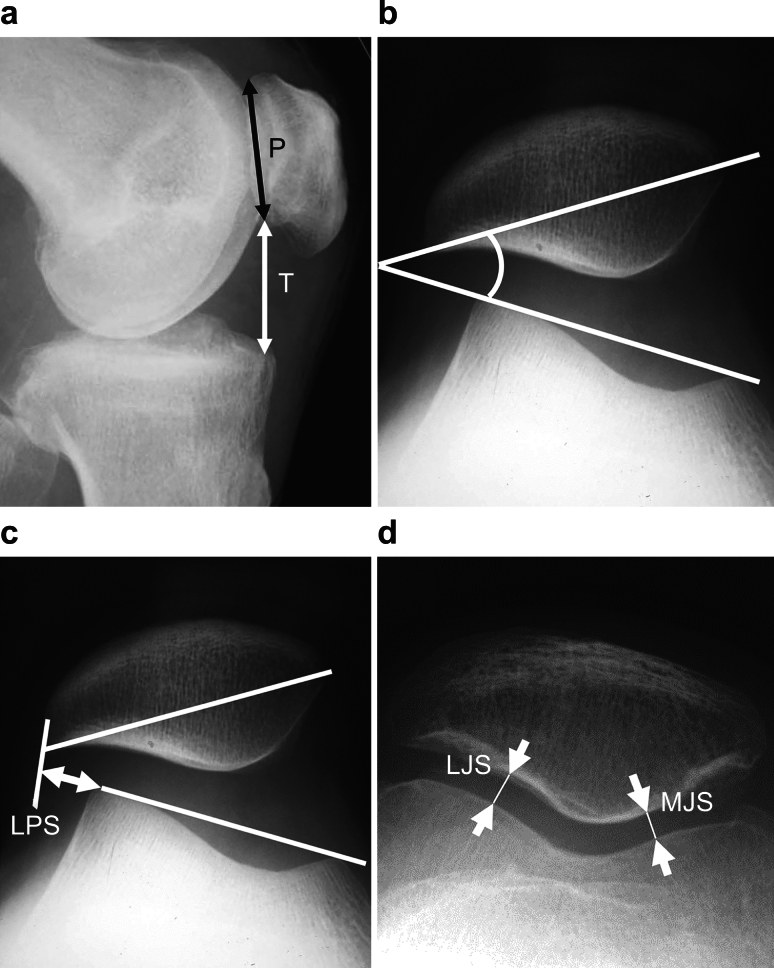
Measurement methods of the patellofemoral joint. **a** Caton–Deschamps index (CDI) = *T*/*P*, where *P* is the patellar articular surface length and *T* is the distance from the anterior angle of the tibial plateau to the most inferior aspect of the patella articular surface. **b** Tilting angle (TA): the angle between the line intersecting the widest bony structure of the patella and the line tangentially passing the anterior surface of the femorl condyle. **c** Lateral patellar shift (LPS): the distance between the summit of the lateral femoral condyle and the point where a line from the lateral edge of the patella perpendicular to the line passes through the summit of the femoral condyles crosses that line. **d** Medial joint space/lateral joint space (MJS/LJS): most narrowed space of the patellofemoral joint. For LPS, MJS, and LJS, the value divided by the width of the patella (p) was also measured to correct the X-ray magnification (LPS/p, MJS/p, LJS/p)

Regarding examination items, the standing femorotibial angle (FTA) and %MA before surgery and at the final follow-up were measured using standing anteroposterior whole-leg radiographs. The tibial correction angle was calculated as the difference in the medial proximal tibial angle (MPTA) after surgery, i.e., when the plate was removed (average time after operation, 15.1 ± 4.5 months), and before surgery. To evaluate the PFJ, the Caton–Deschamps index (CDI) was used for the lateral view in the supine position as an index of patella position, the patellar tilt angle (TA) as an indicator of lateral patella tilt, lateral patellar shift (LPS) as an indicator of lateral patella deviation, and the PFJ joint space width (mm, medial joint space [MJS] and lateral joint space [LJS]) in the supine position and at 30° flexion by skyline view were measured before surgery, at the time of plate removal, and at the final follow-up. Moreover, the values of the LPS, MJS, and LJS divided by the patella width (p) were calculated to correct the X-ray magnification (LPS/p, MJS/p, and LJS/p, respectively) (Fig. 1). The onset of the PFJ OA was judged as positive only when the MJS or LJS had clear narrowing of < 3 mm at the final follow-up (Supplementary Fig. 1). Symptoms derived from the PFJ were evaluated using the hospital for special surgery patellar score (HSS-PS) [12] and the knee injury and OA outcome score patellofemoral subscale (KOOS-PF) [13], which are patient-based assessments. The OA (+) group and the OA (−) roup were compared.

This study was conducted after obtaining approval from the Ethics Committee of Social Medical Corporation Foundation, Sakaide Kaisei General Hospital (reference number: 2022-5). The patients were instructed regarding the study and consent was obtained from them. For statistical processing, statistical processing software minitab14 was used, and paired *t*-test was used to analyze changes over time, the unpaired *t*-test was used to compare the OA (+) group and the OA (−) group, and a risk rate of < 1% was set as significant.

## Results

### Lower extremity alignment

The FTA significantly decreased from 180.8° ± 3.9° before surgery to 170.0° ± 3.6° at the final follow-up. %MA significantly lateralized from 20.8 ± 14.8 before surgery to 66.0 ± 14.6 at the final follow-up. The tibial correction angle was 10.0° ± 3.3° (preoperative MPTA increased from 85.8° ± 2.8° to 95.8° ± 3.2° at the plate removal).

### Patella position

The CDI value was significantly lower upon plate removal (0.68 ± 0.12) and during the final follow-up (0.70 ± 0.10) than that before surgery (0.88 ± 0.12).

### PFJ suitability and joint space

Preoperative values of TA, LPS, and LPS/p significantly decreased upon plate removal; however, no significant difference was found from plate removal to the last follow-up. The preoperative MJS and MJS/p significantly decreased upon plate removal, and a downward trend was observed at the final follow-up. The preoperative LJS and LJS/p significantly decreased upon plate removal and at the final follow-up (Table 1).

**Table 1:** Changes in the patellofemoral joint before and after the OWHTO

	Preoperative		Plate removal		Final follow-up
CDI	0.88	*	0.68	n.s	0.70
TA (degree)	8.74	*	5.25	n.s	4.39
LPS (mm)	6.26	*	4.41	n.s	4.88
LPS/p	0.120	*	0.089	n.s	0.099
MJS (mm)	7.5	*	5.66	p < 0.03	5.09
MJS/p	0.144	*	0.113	p < 0.05	0.104
LJS (mm)	6.56	*	5.82	*	5.25
LJS/p	0.126	*	0.116	*	0.107

*CDI* Caton–Deschamps index, *TA* patellar tilt angle (°), *LPS* lateral patellar shift (mm), *LPS/p* LPS/patella width, *MJS* medial PFJ joint space width (mm), *MJS/p* MJS/patella width, *LJS* lateral PFJ joint space width (mm), *LJS/p* LJS/patella width

Based on these results, it can be stated that the PFJ showed a medial tilt and deviation in the early phase after surgery, and the joint space narrowed over time (Supplementary Fig. 2). The onset of the PFJ OA was observed in 11 MJS and 3 LJS, for an overall rate of 12.4%.

### Alignment differences with and without PFJ OA

No significant difference in lower limb alignment was observed between the PFJ OA (+) and PFJ OA (−) group (Table 2). Moreover, the preoperative alignment of the PFJ was not significantly different (Table 3).

**Table 2:** Comparison of lower limb alignment between the PFJ OA (+) and PFJ OA (−) groups

	PFJ OA (+) (*n* = 14)		PFJ OA (−) (*n* = 99)
Preoperative			
Age	62.9	n.s	64.6
FTA (degree)	181.7	n.s	180.7
%MA (%)	17.7	n.s	21.2
Intraoperative			
Correction angle (degree)	10.9	n.s	9.9
Final follow-up			
FTA (degree)	168.9	n.s	170.2
%MA (%)	71.3	n.s	65.3

**Table 3:** Comparison of the PFJ alignment between the PFJ OA (+) and PFJ OA (−) groups

	PFJ OA (+) (*n* = 14)		PFJ OA (−) (*n* = 99)
Preoperative			
CDI	0.89	n.s	0.88
TA (degree)	10.39	n.s	8.52
LPS (mm)	6.42	n.s	6.23
LPS/p	0.129	n.s	0.119
MJS (mm)	7.15	n.s	7.55
MJS/p	0.142	n.s	0.144
LJS (mm)	6.23	n.s	6.60
LJS/p	0.121	n.s	0.126
Final follow-up			
CDI	0.70	n.s	0.70
TA (degree)	3.03	n.s	4.57
LPS (mm)	4.46	n.s	4.94
LPS/p	0.091	n.s	0.100
MJS (mm)	2.79	*	5.39
MJS/p	0.058	*	0.110
LJS (mm)	4.33	*p* < 0.03	5.37
LJS/p	0.088	*p* < 0.03	0.110

### Symptoms by patient-based evaluation

In all cases, the HSS-PS score was 89.6 ± 11.9 points and the KOO-PF score was 70.1 ± 16.8 points (Table 4). Subsequently, when comparing the PFJ OA (+) group with the PFJ OA (−) group, no significant difference was observed, and the HSS-PS scores for the PFJ OA (+) and OA (−) groups were 91.5 ± 6.6 and 89.3 ± 12.4 points, respectively. For KOOS-PF, the PFJ OA (+) group scored 69.2 ± 16.0 points, whereas the PFJ OA (−) group scored 70.3 ± 17.0 points, and no significant difference was observed. Regarding patient-standing evaluation, no difference between the PFJ OA (+) and the PFJ OA (−) groups was observed (Table 5).

**Table 4:** Details of HSS-PS and KOOS-PS (*n* = 113)

HSS-PS	
VAS (anterior knee pain)	0.86
1.VAS points (50 − 5 × VAS)	45.7
2.Subjective limitations	13.1
3.Objective tenderness	9.0
4.Objective crepitus	13.0
5.Objective QF strength	8.8
Total (sum of 1–5)	89.6
KOOS-PS	
PF01(stiffness after exercise)	0.26
PF02 (pain at rest)	0.48
PF03 (pain-limits activity)	0.14
PF04 (pain rising from sitting)	0.39
PF05 (kneeling pain)	2.45
PF06 (squatting pain)	0.84
PF07 (pain during household activity)	0.86
PF08 (pain at hop/jump)	3.12
PF09 (pain at jog/run)	2.58
PF10 (pain after sports)	1.96
PF11 (pain after modified sports)	0.06
Total (= 100 − (av.PF01 − 11)/4 × 100)	70.13

**Table 5:** Comparison of HSS-PS and KOOS-PF between the PFJ OA ( +) and PFJ OA ( −) groups

HSS-PS	PFJ OA (+) *n* = 14		PFJ OA (−) *n* = 99
VAS (anterior knee pain)	0.55	n.s	0.91
1.VAS points (50 − 5 × VAS)	47.2	n.s	45.4
2.Subjective limitations	13.5	n.s	13.1
3.Objective tenderness	8.5	n.s	9.1
4.Objective crepitus	13.9	n.s	12.8
5.Objective QF strength	8.5	n.s	8.8
Total (sum of 1–5)	91.5	n.s	89.3

KOOS-PF	PFJ OA (+) *n* = 14		PFJ OA (−) *n* = 99
PF01 (stiffness after exercise)	0.31	n.s	0.23
PF02 (pain at rest)	0.46	n.s	0.46
PF03 (pain -limits activity)	0.00	n.s	0.16
PF04 (pain rising from sitting)	0.54	n.s	0.37
PF05 (kneeling pain)	2.38	n.s	2.44
PF06 (squatting pain)	1.15	n.s	0.81
PF07 (pain during household activity)	1.00	n.s	0.85
PF08 (pain at hop/jump)	3.38	n.s	3.08
PF09 (pain at jog/run)	2.77	n.s	2.59
PF10 (pain after sports)	1.54	n.s	1.99
PF11 (pain after modified sports)	0.00	n.s	0.07
Total [= 100 − (av.PF01 − 11)/4 × 100]	69.23	n.s	70.32

The results showed that the postoperative PFJ after OWHTO was tilted and deviated medially early after plate removal, leading to overall joint space narrowing over the long-term and radiographically evident OA in 12.4% of all patients. However, no difference in symptoms was observed during patient-based evaluation between the PFJ OA (+) and PFJ OA (−) groups.

## Discussion

Regarding changes in the PFJ after OWHTO, a medial tilt was observed in the medium-term patients (approximately 5 years postoperatively) [14, 15]. OA was detected in >20% of X-ray images [14], and overall narrowing of the joint space width was predominant on the medial side [15].

The frequency of OA in our study was lower than that reported by Goshima et al. [14]. This can be attributed to the fact that we defined OA as a marked narrowing of the joint space to < 3 mm at the final follow-up, whereas Goshima et al. defined OA as a grade progression according to the Kellgren–Lawrence (KL) classification. When evaluating the progression of OA in the PFJ, we thought that a definition of OA based on actual measurement was more objective than the grade evaluation, such as KL classification to capture slight changes. We did not evaluate the arthroscope findings upon plate removal because they are not accurate and objective in terms of whether the same location as before surgery was observed, whether all joint surfaces were observed, and whether International Cartilage Repair Society (ICRS) grades 2 and 3 can be considered by appearance alone.

Since the medial tilt of the PFJ is observed in OWHTO and DTO [16], it was considered a characteristic change in the valgus of the tibia method. Regarding the onset mechanism of this change in the PFJ, Gaasbeek et al. [17] reported that with an increase in the Q angle, the patella is pulled laterally; as a result of the PF joint pressed laterally, the medial tilt occurs. Elyasi et al. [18] reported that the medial tilt is caused by a change in the contact site between the patella and femoral trochlea, as the tibial tubercle moves distally. Moreover, the pressure to the PFJ increased [19], resulting in OA onset over a long time.

Consequently, no consensus has been established regarding the conditions that cause PFJ OA following OWHTO. According to arthroscopic findings upon plate removal, Lee et al. [10] observed decreased ICRS of the PFJ in 28 of 94 (30%) cases, and compared with the group whose ICRS grade did not worsen by arthroscopic findings, a significant difference in the Kujara score, KOOS pain, activities in daily living, sports and recreational function, and knee related QOL was found. Song et al. [20] also reported that 22 of 50 cases worsened, and a significant difference in Kujara’s anterior knee pain scale score was observed. However, Otakara et al. [9] reported worsening of PFJ in 27 of 57 (47%) cases. Tanaka et al. [8] reported worsening of PFJ in 17 of 52 (32.7%) cases; however, no significant difference in the Knee Society score was noted.

Yoon et al. [21] evaluated X-ray findings in 170 cases at 96.3 months after surgery using KL classification and found 84% of PFJ OA in 11 years. However, anterior knee pain was noted in 5.3% of cases, and the only reoperation case was due to medial femorotibial joint OA recurrence. Although PFJ OA increases over time after OWHTO, no effect on clinical outcome and survival rate in medium- to long-term course was noted.

In this study, in the longer postoperative period, clear joint space narrowing on the X-ray image was judged as OA, and no difference in symptoms in the group with or without OA was found in a patient-based evaluation specialized for PFJ. Although PFJ OA develops after OWHTO, the symptoms are not adversely affected.

Since DTO leaves the tibial tubercle proximally, it does not cause the descent of the patella height and has minor effect on the PFJ. However, serious surgical complications, such as tibial tubercle fracture and risk of tibial shaft fracture distal to the tibial tuberosity osteotomy have been reported [6, 22]. According to Kim et al., complications such as the delayed union of the osteotomy were observed in OWDTO [16]. Considering these risks, whether DTO should be performed due to concerns about its effect on the PFJ or whether OWHTO should be performed with PFJ OA but with mild symptoms and fewer serious complications, remains an issue.

This study has some limitations. First, because the HSS-PS used in the patient-standing evaluation was originally designed for patients with total knee arthroplasty, whether it is appropriate for the evaluation criteria in this study should be considered. Second, only the symptoms at the final follow-up were evaluated, and changes over time could not be evaluated. Third, X-ray evaluation of the PFJ was performed by skyline view; however, in OWHTO, the patella is already lowered distally immediately after surgery, so strictly speaking, the contact area is different before and after surgery. For the former, international evaluation criteria specific to the PFJ must be determined and evaluated before surgery. Fourth, KOOS-PF was used in this study; however, its acceptability must be checked. For the latter, taking a skyline view immediately after surgery and comparing it with subsequent progress will enable a true examination of the changes in the PFJ after OWHTO.

## Conclusions

OWHTO is becoming popular in recent years as a joint-preserving surgery as it is relatively easy to perform and is associated with a shorter post-treatment period. This study demonstrated that in the long-term follow-up after OWHTO, few cases of PFJ OA were evident on X-rays, and no difference in symptoms was found between the groups with or without OA. In future, regarding DTO, which compensates for the weaknesses of OWHTO, we await reports on the improvement of surgical techniques to prevent serious complications and reports on the PFJ after long-term follow-up.

## Supplementary Information

Below is the link to the electronic supplementary material.

Supplementary file1 Definition of patellofemoral joint osteoarthritis onset. (a) Preoperative skyline view. (b) Final skyline view. Significant narrowing of the medial joint space (TIF 433 KB)

Supplementary file2 Schema of the patellofemoral joint change overtime. (a) Preoperative skyline view. Patella tilted and shifted laterally. (b) Skyline view at the time of plate removal. The tilting angle and lateral patellar shift decreased early postoperatively, i.e., pat (TIF 777 KB)

## Data Availability

The datasets generated during and/or analyzed during the current study are not publicly available due to patient confidentiality but are available from the corresponding author on reasonable request.

## Declarations

### Animal Studies

This article does not contain any studies with animal subjects.
