# Supplementary figures and images for: Occurrence of Patellofemoral Joint Osteoarthritis in Long-Term Postoperative Cases of Open-Wedge High Tibial Osteotomy: Differences in Symptoms Based on Patient-Standing Type Evaluation with and Without Patellofemoral Joint Osteoarthritis
Source: Indian J Orthop. 2024 Aug 29;58(10):1411–7. doi: 10.1007/s43465-024-01250-z (PMC11420427; doi:10.1007/s43465-024-01250-z)

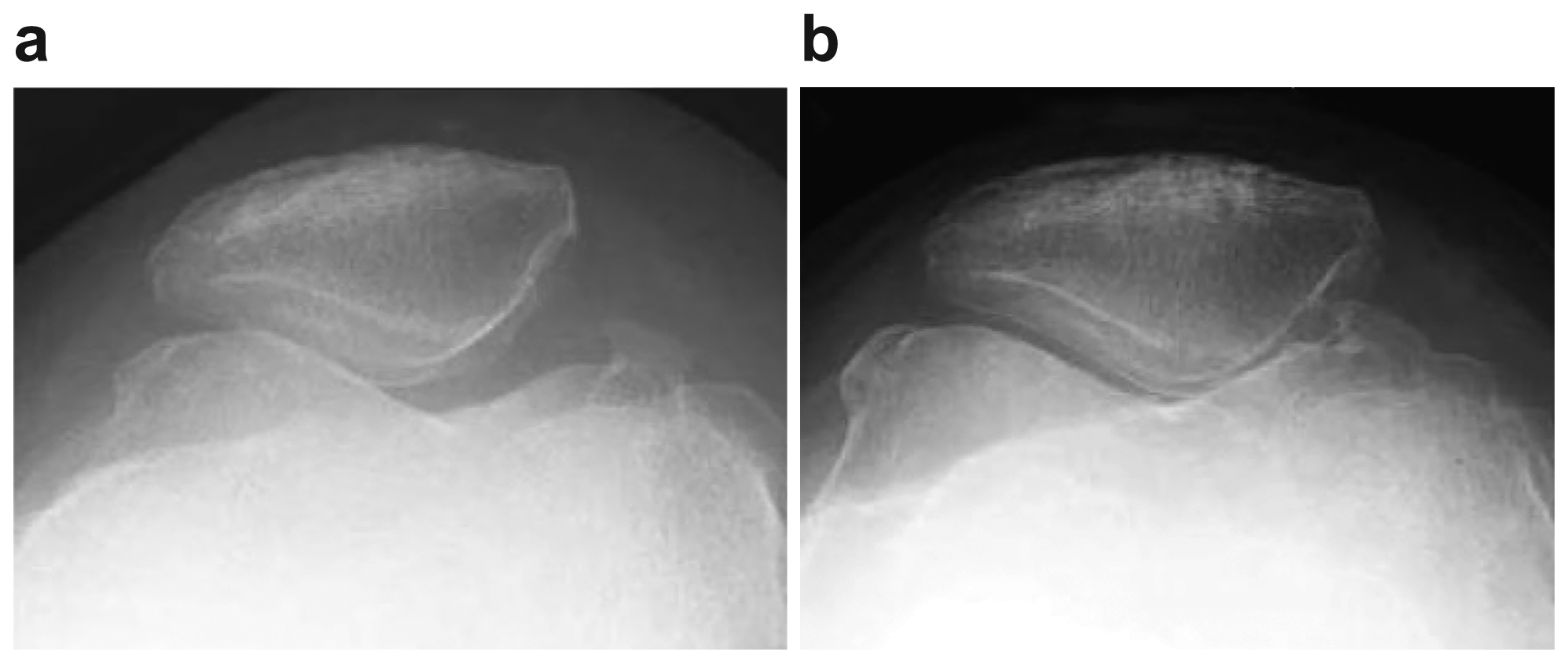

Supplement: Supplementary file 1 — Supplementary file1 Definition of patellofemoral joint osteoarthritis onset. (a) Preoperative skyline view. (b) Final skyline view. Significant narrowing of the medial joint space (TIF 433 KB) [file 43465_2024_1250_MOESM1_ESM.tif]

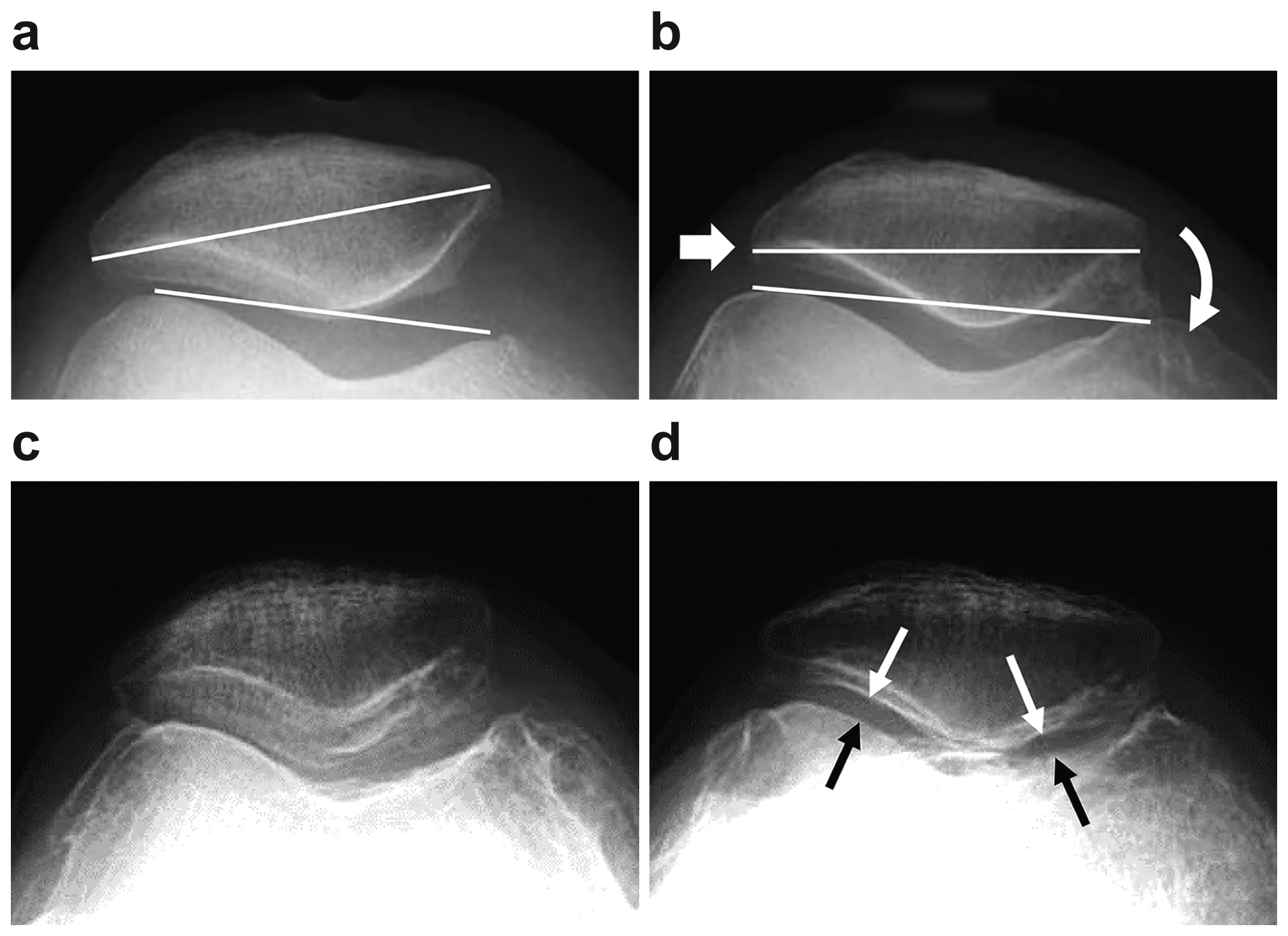

Supplement: Supplementary file 2 — Supplementary file2 Schema of the patellofemoral joint change overtime. (a) Preoperative skyline view. Patella tilted and shifted laterally. (b) Skyline view at the time of plate removal. The tilting angle and lateral patellar shift decreased early postoperatively, i.e., pat (TIF 777 KB) [file 43465_2024_1250_MOESM2_ESM.tif]
